# Supplementary material for: The genome sequence of the Coccidian parasite, Eimeria praecox (Apicomplexa: Eucoccidiorida)
Source: Wellcome Open Res. 2024 Jun 21;9:331. [Version 1] doi: 10.12688/wellcomeopenres.22464.1 (PMC11387943; doi:10.12688/wellcomeopenres.22464.1)
Supplement: Peer review history

# Reviewer report: Reviewer response for version 1

**DOI:** 10.21956/wellcomeopenres.24752.r92009

**Published:** 2024-09-10

**Authors:** Meena Das

**Affiliations:** 1 ICAR Research Complex for NEH Region, Umiam, Meghalaya, India

The objectives and the rationale of the study clearly stated in the present study. The detailed genome assembly of the *Eimeria praecox* Houghton strain is well written and presented in very nice way in the manuscript titled "The genome sequence of the Coccidian parasite, *Eimeria *praecox (Apicomplexa: Eucoccidiorida)," which would be beneficial for further research in this area.

Are sufficient details of methods and materials provided to allow replication by others?

Yes

Is the rationale for creating the dataset(s) clearly described?

Yes

Are the datasets clearly presented in a useable and accessible format?

Yes

Are the protocols appropriate and is the work technically sound?

Yes

Reviewer Expertise:

Veterinary Parasitology, Anthelmintic resistance, Epidemiology of gastrointestinal parasites, Molecular diagnosis of parasites

I confirm that I have read this submission and believe that I have an appropriate level of expertise to confirm that it is of an acceptable scientific standard.

# Reviewer report: Reviewer response for version 1

**DOI:** 10.21956/wellcomeopenres.24752.r92004

**Published:** 2024-09-10

**Authors:** Hanna Hartikainen, Milind Mutnale

**Affiliations:** 1 University of Nottingham, Nottingham, England, UK

The data note presents a chromosome level assembly for a clonal line of Eimeria praecox, a pathogen originally isolated from poultry and maintained in culture since 1966. Despite the expected clonal growth in culture, an alternate haplotype was also discovered. The sequencing quality and coverage obtained appear excellent, and BUSCO score indicates no missing genes. The source material, sequencing and bioinformatics methods are described in good detail.

Are sufficient details of methods and materials provided to allow replication by others?

Yes

Is the rationale for creating the dataset(s) clearly described?

Yes

Are the datasets clearly presented in a useable and accessible format?

Yes

Are the protocols appropriate and is the work technically sound?

Yes

Reviewer Expertise:

Parasitology, evolution, speciation

We confirm that we have read this submission and believe that we have an appropriate level of expertise to confirm that it is of an acceptable scientific standard.

# Reviewer report: Reviewer response for version 1

**DOI:** 10.21956/wellcomeopenres.24752.r92003

**Published:** 2024-08-19

**Authors:** Emanuel Heitlinger

**Affiliations:** 1 Humboldt University Berlin (HU), Berlin, Germany

The manuscript presents the genome assembly of the Houghton strain of *Eimeria praecox. *More specifically it reports on a reference type isolate, which was originally collected in Scotland in 1966 and is now maintained at the Royal Veterinary College as a reference type isolate for the species. The rationale for generating a genome sequence for this important isolate is clear.  

 A clear and concise report summaries the state-of-the art techniques used for the project. The wet-lab techniques and bioinformatics pipelines are clearly described and (especially the latter, for which it is easier to judge) fulfill modern standards of reproducibility.

 The manuscript presents quality metrics which illustrate the (high) quality of the chromosomally-complete genome sequence. The dataset is available through generally established openly accessible databases in standardized format usable for anyone with the relevant knowledge and skills.

Are sufficient details of methods and materials provided to allow replication by others?

Yes

Is the rationale for creating the dataset(s) clearly described?

Yes

Are the datasets clearly presented in a useable and accessible format?

Yes

Are the protocols appropriate and is the work technically sound?

Yes

Reviewer Expertise:

Parasitology, Genomics, Transcriptomics, Evolution

I confirm that I have read this submission and believe that I have an appropriate level of expertise to confirm that it is of an acceptable scientific standard.
